# Design and Formulation of Optimized Microemulsions for Dermal Delivery of Resveratrol

**DOI:** 10.1155/2015/540916

**Published:** 2015-08-31

**Authors:** Vaida Juškaitė, Kristina Ramanauskienė, Vitalis Briedis

**Affiliations:** Department of Clinical Pharmacy, Lithuanian University of Health Sciences, Eivenių Street 4, 50161 Kaunas, Lithuania

## Abstract

The objective of this study was to formulate optimal formulations of microemulsions (MEs) and evaluate their feasibility for delivery of resveratrol into human skin* ex vivo. *Oil-in-water MEs were formulated using surfactant (S) PEG-8 caprylic/capric glycerides and cosurfactant (CoS) polyglyceryl-6-isostearate. Ethyl oleate was used as an oily phase. MEs were formulated using 5 : 1, 6 : 1, and 7 : 1 surfactant and cosurfactant (S : CoS) weight ratios. Pseudoternary phase diagrams were constructed and optimal compositions of MEs were obtained using Design Expert software. Mean droplet size for optimized ME formulations was determined to be 68.54 ± 1.18 nm, 66.08 ± 0.16 nm, and 66.66 ± 0.56 nm for systems with S : CoS weight ratios 5 : 1, 6 : 1, and 7 : 1, respectively. Resveratrol loading resulted in mean droplet size increase. The distribution of droplet size between fractions changed during storage of formulated MEs. Results demonstrated the increase of number of droplets and relative surface area when S : CoS weight ratios were 6 : 1 and 7 : 1 and the decrease when S : CoS weight ratio was 5 : 1. The highest penetration of resveratrol into the skin* ex vivo* was determined from ME with S : CoS weight ratio 5 : 1. It was demonstrated that all MEs were similar in their ability to deliver resveratrol into the skin* ex vivo*.

## 1. Introduction

Skin is a universal barrier protecting living organism from harmful outer factors that are considered as one of the main causes for pathology development. There are numerous skin diseases like erythema and skin cancer or common skin aging that are considered to be caused mainly by UV radiation [1]; thus local delivery of protective agents into the outer layers of the skin could be considered as one of possible solutions. Microemulsions (MEs) are considered as promising drug delivery systems due to their easy formulation, thermodynamic stability, and ability to facilitate delivery of lipophilic and hydrophilic drugs into the skin [2, 3]. MEs consist of aqueous and oily phases, and the disperse system is stabilized by surfactant and cosurfactant components. The composition and the quantities of the MEs components, as well as the included active substances, affect MEs droplets size and its distribution and viscosity that in turn can influence the penetration of the drug into the skin. Surfactant and cosurfactant can act as drug penetration enhancers disrupting the lipids of stratum corneum [3]. It is well established that high concentrations of surfactants can cause skin irritations; accordingly adequate choice of surfactants is a challenge in MEs formulation, and therefore possible effects of qualitative and quantitative composition of surface active components in MEs have to be evaluated, extending their effects on the characteristics of disperse system. PEG-8 caprylic/capric glycerides are nonionic surface active agents and were chosen for stabilization of MEs because of low toxicity and low potential to induce skin irritation [2, 4]. Polyglyceryl-6-isostearate was chosen as cosurfactant in MEs formulation consequently allowing reduction of surfactant amount. The choice of oily phase in MEs is significantly influenced by the lipophilicity of the drug. The lipophilic drug should demonstrate sufficient solubility in the oily phase and is more efficiently delivered into the skin by oil-in-water (O/W) type MEs [5].

Resveratrol was used as a model active substance considering its physiochemical characteristics and possible application in prevention of skin pathology development. Resveratrol is a natural polyphenolic compound, which has demonstrated high antioxidant activity, and can reduce UV causing skin ageing [6, 7]. However the available scientific data show that penetration of polyphenols into the skin is limited due to their poor solubility [8]. Similar solubility problems are intrinsic for resveratrol, and therefore innovative solutions have to be applied with the aim of improving its penetration into skin. For this reason, it is necessary to improve permeation of resveratrol into the skin. Some trials have been performed to formulate MEs and apply them to achieve improved penetration of resveratrol into the skin, and increased penetration has been demonstrated, especially into the dermis [8, 9]. However, further research in the area of possible applications of MEs for drug delivery into the skin is considered relevant as additional data could support better understanding of processes involved in formation of MEs and mechanisms resulting in changes in skin penetration by drug substances.

The aim of this study was to assess possibility of designing optimized formulations of MEs and to evaluate their feasibility for incorporation and dermal delivery of resveratrol into the human skin* ex vivo*. Traditionally MEs development requires extensive testing of numerous experimental formulations. This challenge was attempted to be solved by applying pseudoternary phase diagrams and D-optimal design model (Design Expert software, version 6.0.8; Stat-Ease, Inc., MN, USA) thus minimizing the number of experiments, resources, and time necessary for formulation of optimal MEs.

## 2. Materials and Methods

### 2.1. Materials

Analytical grade transresveratrol was purchased from Sigma-Aldrich Chemie GmBh (Steinheim, Germany). Resveratrol was procured from Naturex (Avignon, France). PEG-8 caprylic/capric glycerides (Labrasol) and polyglyceryl-6-isostearate (Plurol isostearique) were obtained from Gattefosse (Saint-Priest, France) and used as a surfactant and a cosurfactant, respectively. Ethyl oleate, isopropyl palmitate, isopropyl myristate, oleic acid, and olive oil were used as an oily phase in ME and were purchased from Alfa Aesar GmbH & Co KG (Karlsruhe, Germany) and Sigma-Aldrich Chemie GmbH (Steinheim, Germany). Ultrapure water was used as an aqueous phase and was produced using a water purification system Millipore Simplicity (Bedford, USA). Chromatographic grade acetonitrile and acetic acid were obtained from Sigma-Aldrich Chemie GmbH (Steinheim, Germany).

### 2.2. Quantitative Determination of Transresveratrol

HPLC method for quantitative determination of transresveratrol was developed and validated using Agilent 1260 Infinity Capillary LC (Agilent Technologies Inc., Santa Clara, USA) with diode array detector. Separation was performed on Zorbax C18 column (150 × 0.5 mm, 5 *μ*m) using water and acetonitrile (75 : 25, v/v) with 0.5% acetic acid as mobile phase under isocratic conditions. The applied flow rate was 10 *μ*L/min. The temperature of the column was maintained at 25°C. Determination of resveratrol was performed at 306 nm wavelength. Limit of detection (LOD) and limit of quantification (LOQ) were 0.038 *μ*g/mL and 0.125 *μ*g/mL, respectively. The linear range of transresveratrol quantification was established from 0.125 *μ*g/mL to 31.590 *μ*g/mL. The coefficient of determination (*R* ^2^) was 1.000.

### 2.3. Determination of Resveratrol Solubility

An excessive amount of resveratrol was added to 10 g of ethyl oleate, isopropyl palmitate, isopropyl myristate, oleic acid, olive oil (oily phase), or PEG-8 caprylic/capric glycerides (surfactant), and saturated solutions were produced by mixing in shaking incubator (GFL, Germany) for 72 hours at 37 ± 1°C. Undissolved fraction of resveratrol was separated by centrifugation at 6000 rpm for 10 minutes using Eppendorf Centrifuge 5810R (Hamburg, Germany) [4]. Quantity of dissolved resveratrol was determined by HPLC after dilution with ethanol. Resveratrol solubility determination was performed in triplicate.

### 2.4. Pseudoternary Phase Diagram Construction and Optimization

Quantitative ratio of components for oil-in-water (O/W) type MEs was set referring to available scientific data [2, 5, 10]. The suitability of amounts of surfactant, cosurfactant, oily, and aqueous phases for formulation of oil-in-water (O/W) type MEs was confirmed by initial experimental testing, and ranges of quantities are presented in Table 1.

Stability of formulated MEs was evaluated visually at room temperature, and pseudoternary phase diagrams were constructed. The compositions of MEs for optimization were generated by applying D-optimal design model [11, 12], and 14 experimental formulations were produced for compositions containing surfactant and cosurfactant in 5 : 1, 6 : 1, and 7 : 1 ratios. Optimization of experimental MEs was performed setting three criteria as important: (a) MEs droplet size in range from 1 nm to 100 nm; (b) single peak distribution of droplet size; (c) minimum deviation from the mean of the droplet size value.

### 2.5. Formulation of Microemulsions

MEs were formulated using oil titration method at room temperature. Mixtures of surfactant and cosurfactant at different ratios were added to water and mixed by magnetic stirrer IKAMAG C-MAG HS7 (IKA-Werke GmbH & Co.KG, Germany) at 1250 rpm for 10 minutes resulting in opalescent liquid. Oily phase was added by drops under stirring till clear and fluid formulations were obtained. All prepared MEs were stored 24 hours at room temperature for equilibration [13].

#### 2.5.1. Formulation of Microemulsions Containing Resveratrol

Incorporation of resveratrol into MEs was performed by dissolving resveratrol in oily phase and surfactant, as described under Section 2.3 (solubility study of resveratrol in oils and surfactant). Resveratrol containing surfactant and cosurfactant were mixed in different ratios adding calculated amount of water. Then oily phase containing dissolved resveratrol was added by drops, and optimized composition MEs with resveratrol were formulated (MEs-RES). Target content of resveratrol in MEs was set to be 2%. Predominant quantity of resveratrol was incorporated into MEs with surfactant.

### 2.6. Physical Characterization of Microemulsions

Quality of MEs was evaluated by evaluating droplet size, viscosity, pH, conductivity, and oil-in-water (O/W) type. Mean value of droplet size and polydispersity index (PDI) of formulated MEs were measured applying dynamic light scattering technique, using Zetasizer Nano ZS particle size analyzer (Malvern, UK). Measurements were performed at 25°C temperature.

Viscosity was determined using Vibro-Viscometer SV-10 (I&D Company, Limited, Japan), and pH was measured at room temperature, using pH-meter 766 (Knick, Germany).

Microstructure of the system (water-in-oil or oil-in-water) was determined using lipophilic (Sudan I) and hydrophilic (Methylene blue) dyes. Type of microemulsion was confirmed performing conductivity determination at room temperature using conductivity meter (Cond 3110 SET 1, Germany).

### 2.7. Thermodynamic Stability Studies of Microemulsions

Thermodynamic stability studies were applied to evaluate MEs physical stability. The formulations were centrifuged at 3500 rpm for 30 min and homogeneity of the MEs was evaluated. Heating-cooling and freeze-thaw cycles [14] were applied to evaluate MEs thermodynamic stability. MEs were stored at 4°C, 20°C, 32°C, and 45°C for not less than 48 hours in heating-cooling cycle, and at −21°C, 4°C, and 25°C for not less than 48 hours at each temperature regimen in freeze-thaw cycle. Homogeneity of the MEs was evaluated after storage of each of the temperature regimens in both heating-cooling and freeze-thaw cycles.

### 2.8. *Ex Vivo* Resveratrol Skin Permeation Studies

Caucasian woman (age range 25–40 years) abdominal skin was obtained from the Department of Plastic and Reconstructive Surgery, Hospital of Lithuanian University of Health Science, Kauno Klinikos. The studies of transdermal permeation were performed in accordance with the permission of Kaunas regional Biomedical Research Ethics Committee. The skin was kept at −20°C for less than 6 months before use in the study. Skin permeation* ex vivo *studies (*n* = 6) were performed using Bronaugh type flow-through diffusion cells with full thickness human skin. Diffusional area in the cell was 0.64 cm^2^. The cells were equilibrated for 12 hours using thermostated circulating water bath Grant GD120 (Grant Instruments Ltd., UK) at 37°C temperature. Peristaltic pump (Masterflex L/S) with a multichannel pump head (Cole-Parmer Instrument Co., USA) was used to circulate acceptor phase, consisting of 0.9% NaCl to create physiological tonicity and 0.005% NaN_3_ to prevent microbial growth. Infinite dose of resveratrol in solvents or MEs (200 mg) was applied on the outer surface of the skin. Resveratrol containing media was removed after 24 h, and skin surface was washed twice with 500 *μ*L 96% ethanol and 3 times with 0.9% NaCl. Epidermis was peeled off from dermis after application of preheated metal surface (approx. 60°C) for 1-2 seconds on epidermis side [15–17]. Extraction of resveratrol from epidermis and dermis was performed using methanol (HPLC grade) under sonication.

### 2.9. Statistical Analysis

MEs optimization was performed using experiment planning software Design-Expert 6.0.8, D-optimal design model. Statistical analysis of experimental data was performed using SPSS software (version 19.0) and Microsoft Office Excel 2013. One way ANOVA (Turkey's Honestly Significant Difference criteria) was used for statistical analysis. Spearman's rank coefficient was used for correlation analysis. Statistically significant difference was determinate when value of *P* < 0.05.

## 3. Results and Discussion

The evaluation of solubility of transresveratrol revealed significantly higher solubility in surfactant if compared to oily phases used in ME formulation (Table 2). Results demonstrated that solubility of resveratrol in surfactant was 83.85 ± 7.66 mg/g, while determined solubility of resveratrol in tested oily phases showed that it has not exceeded 0.5 mg/g thus being up to 160-fold lower if compared to PEG-8 caprylic/capric glycerides. No statistically significant difference (*P* > 0.05) was determined for solubility of resveratrol in ethyl oleate (0.42 ± 0.07 mg/g), isopropyl palmitate (0.41 ± 0.04 mg/g), and isopropyl myristate (0.49 ± 0.05 mg/g).

Ethyl oleate was chosen as an oily phase for formulation of MEs as available data demonstrate maximum efficiency of PEG-8 caprylic/capric glycerides solubilizing capacity of ethyl oleate instead ofisopropyl myristate [18].

With the reference to applied approaches for formulation of MEs the S : CoS weight ratios 1 : 1, 2 : 1, 3 : 1, 5 : 1, 6 : 1, and 7 : 1 were chosen and used in 40, 50, 60, and 70% in MEs [8, 19]. Stable MEs were formed using S : CoS weight ratios 1 : 1, 2 : 1, and 3 : 1 when the total quantity of surface active agents was 60% and higher. High concentrations of surface active agents in MEs are recognized to represent main risk factor for skin irritation and thus should be avoided in formulation of products for topical application [3, 18, 20]. These S : CoS weight ratios were not chosen for further testing because of insufficient stability of MEs at concentrations of surface active agents lower than 60%. Separation of phases in MEs was observed when the applied S : CoS (weight ratios 5 : 1, 6 : 1, and 7 : 1) quantity was 40%, thus it was decided to increase gradually the quantity of surface active agents till formation of stable MEs. Formulations of MEs retained stability when S : CoS weight ratio was 5 : 1, and concentration of S : CoS was higher than 45% content. Phase separation in MEs was observed after 24 h when S : CoS weight ratio was 6 : 1 or 7 : 1 (aqueous phase 40%, oily phase 15%). Increase of S : CoS content to 46% resulted in formation of stable MEs.

The design of experiment was applied to define the minimum number of experimental compositions of O/W type MEs in the defined region of pseudoternary phase diagram. The input of limit values for MEs forming components (Table 1) produced 14 experimental formulations for compositions containing S : CoS in 5 : 1, 6 : 1, and 7 : 1 weight ratios (Tables 3, 4, and 5).

**Figure 1:**
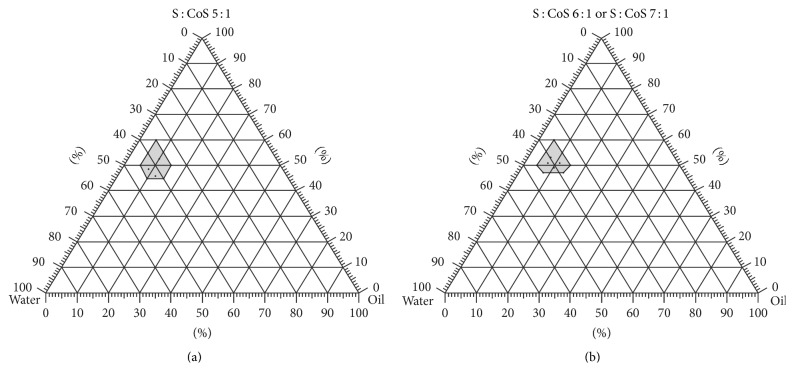
Pseudoternary phase diagrams of ranges of stable MEs when PEG-8 caprylic/capric glycerides and polyglyceryl-6-isostearate are used at ratios 5 : 1 (a), 6 : 1, and 7 : 1 (b).

The gray area in Figure 1 represents range where 14 software generated compositions of MEs were located and formation of stable MEs was expected.

Formulated MEs were evaluated regarding mean droplet size, single peak distribution of droplet size, and minimum deviation from the mean of droplet size value (Tables 3, 4, and 5). Measurements of MEs' droplet size of 14 experimental formulations demonstrated that droplet size mean value was in the range 49.14–86.98 nm, when S : CoS weight ratio was 5 : 1. Smaller mean values of droplet size were determined for MEs containing S : CoS in ratio 6 : 1 (48.51–79.51 nm), and for MEs containing S : CoS in ratio 7 : 1 (47.67–79.94 nm). This supports assignment of produced disperse systems to MEs [20]. Characterization of formulated MEs was performed by evaluating homogeneity of the disperse phase in the systems. Presence of two-peak droplet distribution pattern was determined in certain formulations of experimental MEs with S : CoS weight ratio 5 : 1. The PDI values of MEs with single peak distribution of droplet size were relatively low (0.248–0.379), indicating the homogeneity of disperse phase in MEs formulations [14].

Thermodynamic stability studies demonstrated satisfactory stability of experimental formulations as all prepared MEs remained transparent, no creaming, cracking, precipitation, or phase separation has been observed, and consequently were included in optimization program.

Suitability and significance of mathematical model were confirmed by statistical parameters (*R* ^2^, adjusted *R* ^2^, predicted *R* ^2^, standard deviation, and predicted residual error sum of squares), and optimization of formulations was performed applying desirability function. Desirability of parameter ranged from 0 to 1, with values verging towards 1 representing the most desired.

**Figure 2:**
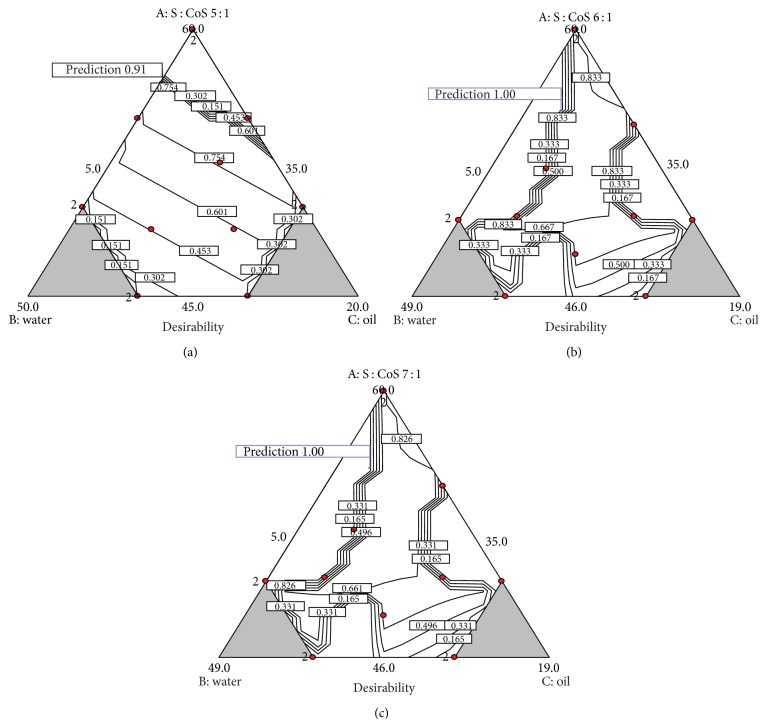
Areas of optimal composition of MEs with highest desirability parameter for compositions with ratios of PEG-8 caprylic/capric glycerides and polyglyceryl-6-isostearate, 5 : 1 (a), 6 : 1 (b), and 7 : 1 (c).

All optimal compositions of MEs, used for further investigations, are demonstrated in Figure 2. The highest desirability value of 0.91 was identified for single optimal composition of MEs with S : CoS weight ratio 5 : 1, and this composition was accepted for further testing (Figure 2(a)). Five and seven optimal compositions with desirability value of 1 were produced for MEs with S : CoS ratios 6 : 1 and 7 : 1, respectively. Therefore one optimal composition for MEs with S : CoS ratios 6 : 1 and 7 : 1 was selected considering the ability to incorporate high quantity of oily phase as an additional factor ensuring high loading of resveratrol (Figures 2(b) and 2(c)). Selected optimal compositions of MEs are presented in Table 6.

The concept of the model was confirmed by formulating and evaluating optimized MEs with predefined quantitative composition. The O/W type of optimized MEs' was confirmed by using lipophilic (Sudan I) and hydrophilic (Methylene blue) dyes. Comparison of theoretically designed characteristics and results of determination of droplet characteristics in formulated MEs is presented in Table 7.

The determined mean droplet size and standard deviation were smaller for OPT-ME 5 : 1 if compared to designed values by 5.7% and 7.04%, respectively. For OPT-ME 6 : 1 and OPT-ME 7 : 1 determined droplet size was, respectively, 1.3% and 2.97% higher when compared to theoretical model. Standard deviations for mean droplet size of designed and determined values for OPT-ME 6 : 1 differed by 1.2% and for OPT-ME 7 : 1 differed by 0.25%, and could be considered as insignificant. The results indicated suitability of applied theoretical approach for modelling of MEs; consequently the same approach and compositions of MEs were used for formulation of MEs containing a model active substance resveratrol. Resveratrol was dissolved in surfactant and oily phase, thus minimizing possible redistribution of resveratrol between phases of MEs and achieving high incorporated quantities. Resveratrol was incorporated in optimized MEs with S : CoS weight ratios 5 : 1 (ME-RES 5 : 1), 6 : 1 (ME-RES 6 : 1), and 7 : 1 (ME-RES 7 : 1). Physical characterization of MEs was performed to detect possible effects of resveratrol inclusion into disperse phase of MEs (Table 8). Conductivity, viscosity, and pH measurements were performed 24 hours after formulating MEs. Determined average conductivity for all tested MEs was close to 100 *μ*S/cm, confirming that formulated MEs were of O/W type [11, 21, 22].

The viscosity of OPT-ME formulations varied between 53.95 and 62.80 Pa·s, and these were similar to the published data for same type of MEs [20, 21]. The inclusion of resveratrol into formulations of optimal MEs caused increase of disperse system viscosity.

The pH of all formulated optimized MEs varied between 7.01 and 7.15. MEs-RES were compared with OPT-MEs and it was determined that pH value of MEs-RES is lower but still was in physiologically acceptable range [23].

Characterization and stability evaluation of MEs were performed by measuring droplet size distribution 24 hours, 48 hours, and 1 week after formulation. MEs-RES remained transparent; no signs of phase separation, cracking, or creaming were identified. The increase of mean value of droplet size was 41.3% for ME-RES 5 : 1, 37.4% for ME-RES 6 : 1, and 38.6% for ME-RES 7 : 1 when compared to OPT-MEs, and it could be related to incorporation of resveratrol. PEG-8 caprylic/capric glycerides demonstrated relatively high solubility of resveratrol (Table 2). Therefore the increase of droplet size could be associated with a predominant location of resveratrol in the interfacial film and so affecting the function of surfactants in MEs.

**Figure 3:**
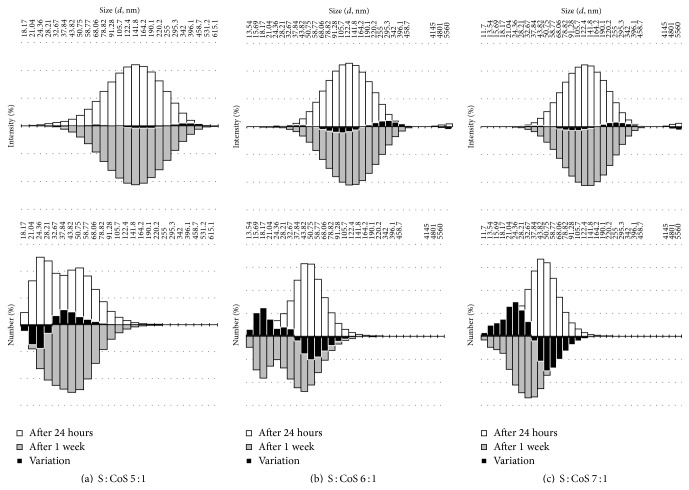
Changes in droplet size distribution in MEs during storage.

Droplet size measurements in resveratrol containing MEs revealed existence of two-peak droplet distribution in ME-RES 6 : 1 and ME-RES 7 : 1 24 and 48 hours after formulation. No droplets in bigger size range, represented by smaller peak, were determined after 1 week. The changes in the disperse phase characteristics were studied by evaluating alterations of the droplet size fractional composition in MEs. Droplet fractional distribution according to light scattering intensity and droplet number is presented in Figure 3.

Droplet size in ME-RES 5 : 1 varied between 21.04 and 458.70 nm 24 hours after formulation, and similar droplet size distribution profile was determined after 48 hours. Droplet size changes were observed in 1 week after formulation of MEs as fractions of droplets with diameter less than 21.04 nm and bigger than 531.20 nm have been no longer determined (Figure 3(a)). The dominating fraction amount of the droplets remained the same; however, minor redistribution of dispersion phase between fractions was identified as determined by intensity measurements. Interfacial surface area change was considered as the potential indicator for stability of disperse system; consequently relative values were calculated for 1 g of MEs. Measurements performed at 24-hour point and after 1 week of storage of MEs at room temperature and comparing the number of droplets per 1 g and their relative surface area revealed decrease by 24.3% and 8.4%, respectively, indicating the increasing internal stability of the disperse system. Droplet size measurements in ME-RES 6 : 1 and ME-RES 7 : 1 after 24 and 48 hours demonstrated the presence of two-peak distribution of droplets when evaluating the system by intensity. Evaluation of the system by number of droplets showed no droplets in the range above 255 nm thus confirming small number of droplets existing in that size range. Peak ranges of droplet sizes when evaluated by intensity were from 24.36 nm to 458.70 nm, and from 4145 nm to 5560 nm, showing wide gap between sizes of present droplets. Similar droplet distribution profile was determined after 48 hours demonstrating presence of the second peak in bigger diameter droplet range for both ME-RES 6 : 1 and ME-RES 7 : 1. The second peak was not determined after 1 week, as redistribution of droplets between fractions occurred that resulted in appearance of smaller diameter droplet fraction (Figures 3(b) and 3(c)). The total droplet number increased by 54.6% and relative surface area in the disperse system increased by 23.8% in ME-RES 6 : 1 when data from 24 hours and 1 week measurements were compared. Similar changes were determined in ME-RES 7 : 1 disperse system: the total droplet number and relative surface area increased by 72.8% and 30.5%, respectively.

Stability of formulated MEs was evaluated by measuring droplet fraction distribution after 24 hours and 1 week after formulation. The decrease of droplet number for ME-RES 5 : 1 in the droplet size range 18.17–30.44 nm was observed to reach 36.49% when data of 1 week stability evaluation was compared to 24-hour stability testing results. Conversely, the droplet number in the size range 30.44–54.76 nm increased by 17.9% after same time interval. Opposite tendencies were determined for ME-RES 6 : 1, and ME-RES 7 : 1, as number of droplets in the droplet range from 40.83 to 73.44 nm decreased by 29.29% and by 41.8%, respectively, when 1-week data were compared to 24-hour testing results. Similar increase of droplet number in fine fractions of ME-RES 6 : 1 and ME-RES 7 : 1 was determined. The number of droplets increased by 67% in fractions from 14.62 nm to 30 nm for ME-RES 6 : 1, and for ME-RES 7 : 1 the number of droplets increased by 78.17% in fractions from 16.93 nm to 30.44 nm when results of 1 week were compared to those of 24 hours. The changes in the fractional composition of MEs demonstrated liability of the formulated disperse systems to acquire most favorable physical stability status and changes in composition of interfacial film after incorporation of resveratrol appropriately affecting packing pattern of surfactants at the interface.

**Figure 4:**
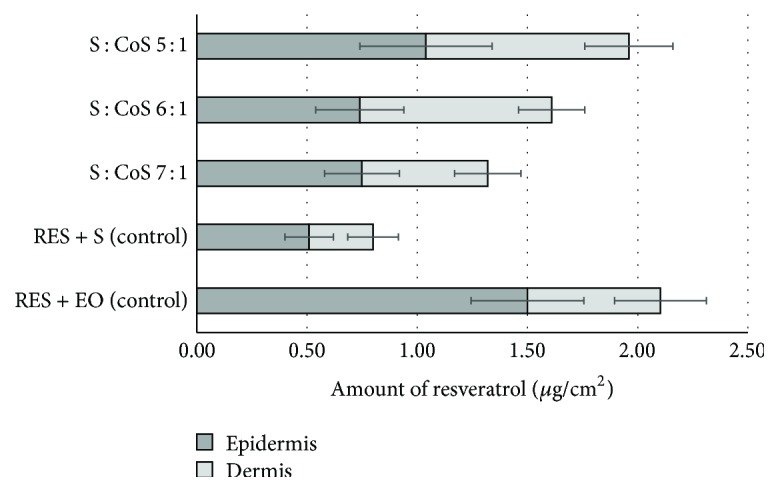
Cumulative amount of resveratrol in epidermis and dermis after application of formulations for 24 hours.

The penetration of resveratrol into the human skin* ex vivo* was determined to evaluate presumptive formulation effects on the bioavailability of active ingredient. The results of determination of resveratrol penetration into skin layers after application of formulated MEs and control samples for 24 hours are presented in Figure 4.

Results demonstrated that highest penetration of resveratrol into the skin* ex vivo* (1.96 ± 0.41 *μ*g/cm^2^) was determined when ME with S : CoS weight ratio of 5 : 1 was applied. The amount of resveratrol in the skin after application of ME-RES 6 : 1 was 1.62 ± 0.27 *μ*g/cm^2^, and only 1.31 ± 0.27 *μ*g/cm^2^ of resveratrol in the skin accumulated after application of ME-RES 7 : 1. No statistically significant (*P* > 0.05) effect of S : CoS weight ratios in MEs on penetration of resveratrol into epidermis was established comparing penetration of resveratrol from MEs, when S : CoS weight ratios were 5 : 1, 6 : 1, and 7 : 1. Statistically significant (*P* < 0.05) difference was established between penetration of resveratrol into dermis from ME with S : CoS weight ratio 5 : 1, and MEs with S : CoS weight ratios 6 : 1 or 7 : 1. Results established the existing statistically significant (*P* < 0.05) inverse correlation (*r* = −1) between S : CoS weight ratios and resveratrol penetration into the skin. This correlation revealed that increases of S : CoS weight ratio in MEs resulted in decreased penetration of resveratrol into the skin. Correlation between droplet size and resveratrol penetration into the skin was not established. Delivery of resveratrol by applying formulated MEs resulted in efficient migration of resveratrol through epidermis into the deeper dermis, and the pattern of distribution of active component in the skin layers* ex vivo* differed from those observed after application of resveratrol in PEG-8 caprylic/capric glycerides or ethyl oleate used as control. When MEs were used for resveratrol delivery to the skin, distribution ratio of resveratrol in epidermis and dermis ranged from 0.9 to 1.3 thus indicating relatively higher quantities of resveratrol in hydrophilic dermis.

The penetration of resveratrol into the skin* ex vivo* from PEG-8 caprylic/capric glycerides (S) and ethyl oleate (EO) was determined to evaluate possible penetration modifying effects of the excipients. The amount of resveratrol in the skin was 2.1 ± 0.36 *μ*g/cm^2^ with ethyl oleate containing 0.031% resveratrol (RES + EO) and 0.8 ± 0.20 *μ*g/cm^2^ when surfactant with 5.2% resveratrol (RES + S) was applied for 24 hours. The application of resveratrol dissolved in ethyl oleate resulted in predominant accumulation of active substance in epidermis and limited diffusion to dermis. Distribution ratio of resveratrol between epidermis and dermis was 2.5 when ethyl oleate was used as carrier, indicating that up to 70% of applied resveratrol dose remained in epidermis. Therefore ethyl oleate should not be considered as efficient carrier for resveratrol. Ability of ethyl oleate to disturb and fluidize lipid structures in stratum corneum and lipophilicity of resveratrol (log⁡*P* = 3.1) can be used to explain penetration and accumulation of resveratrol in epidermis [24]. Similar distribution pattern but lower quantities of resveratrol in skin layers were determined after application of PEG-8 caprylic/capric glycerides.

The results demonstrated ability of formulated MEs to alleviate penetration of resveratrol into the deeper layers of the skin without developing high concentration gradient between epidermis and dermis, and these findings support the suitability of MEs for enhancing the delivery of resveratrol to the dermis [8, 9]. MEs formulated using PEG-8 caprylic/capric glycerides and polyglyceryl-6-isostearate could be used for targeted delivery of lipophilic active substances into dermis.

## 4. Conclusions

MEs represent a spontaneously forming disperse system, and the characteristics of disperse phase are affected by the nature and ratio of the components. Attempts to formulate MEs with designed droplet characteristics present a development challenge as multiple interactions of system components have to be taken into consideration. Design of experiment approach was applied for reducing the number of experimental formulations and modeling of optimal compositions of MEs with predefined quality characteristics. Validity of the model was verified by formulation MEs and their characterization. Attempts to incorporate resveratrol into optimal MEs resulted in increase of mean droplet size up to 41.3% thus demonstrating possible effects of active ingredients on disperse system characteristics if they have predisposition to accumulate in the interfacial film. Redistribution of droplet size within fractions was determined while analyzing the processes of droplet size increase, thus indicating possible changes in the interfacial film components' packing pattern. Increasing penetration of resveratrol into the skin* ex vivo* was related to the increasing amount of cosurfactant polyglyceryl-6-isostearate. Effect of droplet size on permeability of resveratrol was not evaluated as formulation of MEs with different droplet size required to make changes MEs composition.

## Figures and Tables

**Table 1:** Quantitative ranges of components in formulated microemulsions.

ME components	Function	Amount, %
Water	Aqueous phase	35–45

Ethyl oleate	Oily phase	5–15

PEG-8 caprylic/capric glycerides and polyglyceryl-6-isostearate	Surfactant and cosurfactant	45–60

**Table 2:** The solubility of transresveratrol in oils and surfactant (*n* = 3).

Phase type	Excipient	Solubility (mg/g)
Oily	Isopropyl myristate	0.49 ± 0.05
	Isopropyl palmitate	0.41 ± 0.04
	Ethyl oleate	0.42 ± 0.07
	Oleic acid	0.07 ± 0.01
	Olive oil	0.20 ± 0.01

Surfactant	PEG-8 caprylic/capric glycerides	83.85 ± 7.66

**Table 3:** Compositions of MEs with S : CoS 5 : 1, generated by D-optimal design model and measured responses.

Number	Components of MEs %			Mean droplet size, nm	Standard deviation, nm	Number of peaks
	S : CoS 5 : 1	Water	Oil			
1	52.5	37.5	10.0	70.50	54.98	1
2	50.0	45.0	5.0	49.14	72.82	1
3	45.0	45.0	10.0	50.29	81.27	2
4	60.0	35.0	5.0	86.98	50.90	1
5	50.0	35.0	15.0	79.16	63.49	1
6	45.0	40.0	15.0	74.22	88.06	2
7	55.0	40.0	5.0	61.16	50.83	1
8	48.8	42.5	8.8	67.80	71.23	1
9	48.8	38.8	12.5	57.36	66.28	1
10	55.0	35.0	10.0	79.45	46.94	1
11	60.0	35.0	5.0	84.45	63.02	1
12	50.0	35.0	15.0	81.31	68.77	1
13	50.0	45.0	5.0	51.09	92.49	1
14	45.0	45.0	10.0	50.60	79.10	2

**Table 4:** Compositions of MEs with S : CoS 6 : 1, generated by D-optimal design model and measured responses.

Number	Components of MEs %			Mean droplet size, nm	Standard deviation, nm	Number of peaks
	S : CoS 6 : 1	Water	Oil			
1	60.0	35.0	5.0	78.08	46.55	1
2	46.0	39.0	15.0	76.16	77.73	2
3	50.0	45.0	5.0	48.78	55.01	1
4	50.0	35.0	15.0	79.51	64.93	1
5	46.0	45.0	9.0	48.54	80.31	1
6	52.7	39.9	7.4	60.47	51.16	1
7	55.0	35.0	10.0	75.89	46.95	1
8	48.2	40.9	10.9	59.65	67.01	1
9	50.2	37.4	12.4	69.01	61.74	1
10	50.2	42.4	7.4	55.36	58.29	1
11	50.0	45.0	5.0	48.51	54.26	1
12	46.0	39.0	15.0	75.17	83.72	2
13	60.0	35.0	5.0	77.51	47.03	1
14	46.0	45.0	9.0	48.74	77.03	1

**Table 5:** Compositions of MEs with S : CoS 7 : 1, generated by D-optimal design model and measured responses.

Number	Components of MEs %			Mean droplet size, nm	Standard deviation, nm	Number of peaks
	S : CoS 7 : 1	Water	Oil			
1	60.0	35.0	5.0	76.12	45.56	1
2	46.0	39.0	15.0	77.69	77.72	2
3	50.0	45.0	5.0	47.68	51.93	1
4	50.0	35.0	15.0	79.94	64.64	1
5	46.0	45.0	9.0	47.67	71.18	1
6	52.7	39.9	7.4	59.00	50.04	1
7	55.0	35.0	10.0	74.88	45.91	1
8	48.2	40.9	10.9	58.89	65.12	1
9	50.2	37.4	12.4	68.01	56.20	1
10	50.2	42.4	7.4	54.07	55.33	1
11	50.0	45.0	5.0	47.75	51.11	1
12	46.0	39.0	15.0	77.19	80.39	2
13	60.0	35.0	5.0	75.79	43.84	1
14	46.0	45.0	9.0	47.95	74.35	1

**Table 6:** Optimal compositions of microemulsions (OPT-MEs) with different S : CoS weight ratios.

Sample	S : CoS amount, %	Water amount, %	Oil amount, %
OPT-ME 5 : 1	57	38	5
OPT-ME 6 : 1	55,5	37,9	6,6
OPT-ME 7 : 1	56	37,6	6,4

**Table 7:** Comparison of characteristics of MEs (*n* = 3).

Sample	Mean droplet size, nm		Standard deviation, nm		Number of peaks
	Designed value	Determined value	Designed value	Determined value	
OPT-ME 5 : 1	72.66	68.54 ± 1.18	57.33	53.29 ± 6.09	1
OPT-ME 6 : 1	65.24	66.08 ± 0.16	45.43	44.87 ± 2.26	1
OPT-ME 7 : 1	64.68	66.66 ± 0.56	43.62	43.73 ± 2.28	1

**Table 8:** Characteristics of optimized (OPT-MEs) and resveratrol containing MEs (MEs-RES).

	OPT-ME 5 : 1	ME 5 : 1 2% RES	OPT-ME 6 : 1	ME 6 : 1 2% RES	OPT-ME 7 : 1	ME 7 : 1 2% RES
pH	7.01 ± 0.06	6.95 ± 0.01	7.15 ± 0.04	6.94 ± 0.01	7.11 ± 0.01	6.96 ± 0.01
Conductivity, *µ*S/cm	95.55 ± 0.07	93.50 ± 0.14	84.45 ± 0.07	83.85 ± 0.35	74.45 ± 0.07	72.95 ± 0.07
Viscosity, mPa·s	62.80 ± 0.14	67.30 ± 1.27	53.95 ± 0.92	61.65 ± 1.48	54.35 ± 1.77	59.35 ± 1.48
Particle size, nm	68.54 ± 1.18	116.83 ± 0.23	66.08 ± 0.16	105.6 ± 0.30	66.66 ± 0.56	108.63 ± 0.68

Measurements made in triplicate (*n* = 3).
